# The Journal and the Association

**Published:** 1894-04

**Authors:** 


### EDITORIAL DEPARTMENT.

F. E. DANIEL, M. D., Editor.
S. E. HUDSON, M. D., Managing Editor.
A. J. SMITH. M. D., Galveston, Associate Editor.

EDITORIAL STAFF:

PROF. J. E. THOMPSON, M. D., Texas Medical College, Galveston; Surgery.
PROF. WM. KEILLER, M. D., Texas Medical College, Galveston; Obstetrics and Gynecology.
PROF. DAVID CERNA, M. D., Texas Medical College, Galveston; Therapeutics.
PROF. A. J. SMITH, M. D., Texas Medical College, Galveston; Medicine
DR. ISADORE DYER, Tulane University; Dermatology.
DR. R. H. L. BIBB, Saltillo, Mexico; Foreign Correspondent.
DR. ROBT. MORRIS, Charity Hospital, N. O.; Clinical Reports

### THE JOURNAL AND THE ASSOCIATION.

That the TEXAS MEDICAL JOURNAL is the staunch friend and well wisher and supporter of the State Medical Association has been a matter of record many years, sufficiently demonstrated on every occasion. It has been its promoter, defender and champion. We early began to advocate "organization," and for ten years, as our pages show, have steadily insisted upon it, as a means to ends; in fact, in those words are declared our mission and aim, in our first prospectus.

Because, at times the JOURNAL has severely criticised certain action—and at other times failure of action on the part of the Association; because we have brought to the attention of that body certain things that were going on, which constituted a reproach to the very name of medicine, and other things which threatened its peace and its very existence, and in consequence thereof, agitation, and for a time strife was produced, it is no more to be assumed that we were *unfriendly* towards that body, than is a parent who, for his child's good, corrects errors in his character, and checks tendency to evil. It was because the JOURNAL loved the Association, and to save it, that we followed the course we have done all these years. Whenever anything has come to the knowledge of the JOURNAL, calculated, in our judgment, to detract from the honor and dignity of the profession, or which threatened the Association's integrity, the JOURNAL has fearlessly condemned it, and pointed out the proper course to be pursued.

After all this agitation, the result is salutary. Like wine which has undergone fermentation, all the dregs have been precipitated, and the clear liquor, strong and wholesome, remains, in a state of undisturbed serenity. All is now peace; and henceforth, our hope is, that there shall "not a wave of trouble roll across its peaceful breast." The Association has undoubtedly gone through a long period of unrest; but now it is serene and strong; there is not a disturbing element in its organization, and it can proceed to business. What that business is—it would be an insult to the intelligence of every reader to specify; it is self-evident. Let members look around them and see what the State Associations are doing in other States, Ohio, particularly, and undismayed by past failures, renew their efforts; for one thing,—to secure much needed medical legislation.

It has been thrown up to the Association by unfriendly parties that its membership does not embrace ten per cent. of the physicians of the State. While that is true, it is no reflection on the worth or merit of the organization. Texas is a very large State, and for the greater part of its area. is sparcely settled. The majority of physicians are so situated that they would have to travel, perhaps horseback or in a buggy, an inconvenient distance to attend its meetings. The time will never come, for this reason, when the Association will include a much larger per cent. of the physicians than it does now. Our observation, extending over a period of twelve years, is that at each meeting most of the physicians in easy access of the place of meeting, join. The next year and the next, the meetings will be held at points out of their reach, and in that way the same physicians—with perhaps fifty exceptions,—do not attend all meetings. When the convention is held at San Antonio, Houston or Galveston, there is a large attendance of those accessible,—for this part of the State is thickly populated with doctors; when we meet at Tyler or Paris, those accessible join, and the meeting is largely made up of physicians from that section,—few of those who joined at Galveston or Houston or San Antonio, putting in an appearance. And, as the meetings are held about, first in one section of the State, then in another, the majority of physicians find it impossible to attend every meeting, and thus they lose interest and drop out. The membership is thus changeable, not fixed.

As it has been said that the Association is disintegrating, we have compiled the following table as showing the movement of the membership, and illustrating our position. If all who have at various times been members are counted, it will be seen that the discrepancy is not so great:

| | |
|---|---|
| In 1884 there were 280 members | 280 |
| In 1884, admitted at Belton | 89 |
| In 1885, at Houston | 39 |
| In 1886, at Dallas | 87 |
| In 1887, at Austin | 52 |
| In 1888, at Galveston | 32 |
| In 1889, at San Antonio | 58 |
| In 1890, at Fort Worth | 48 |
| In 1891, at Waco | 44 |
| In 1892, at Tyler | 35 |
| In 1893, at Galveston | 37 |
| Making a total of | 801 |
| 1894, the roll at present numbers | 386 |
| Showing a loss of | 415 |

Against a gain of 521, and a net gain of 106; or about 38 per cent. permanent gain.

Is there a remedy for this? Is there any way in which a larger number of the physicians can be gotten into and held to membership? We might try a change of policy with regard to our annual meetings. Instead of skipping about from south to north, and from east to west, all over the State, in the effort to accommodate all—and in effect inconveniencing the majority of members, perhaps,—we might have a fixed place of meeting, say at the capital. That there are objections to this, which can be urged with force, we are aware; but it would, perhaps, have the effect of holding together and securing, at every meeting, a larger number,—and representing a larger area—than is the case under the present plan.

The JOURNAL urges every physician, who reads this, to make his arrangements to get off the last week in April and run over to the capital to the big meeting. Austin will have on her loveliest spring attire in April. Strawberries will be ripe, and roses in bloom. Our sweet south breezes will be laden with the breath of violets wafted from the "violet crown" of the adjacent cedarclad hills, and all will be lovely. There will doubtless be an unusually large attendance, and physicians will have a rare chance for professional reunion and pleasant social intercourse; of extending their acquaintance and broadening their views of many things. All work and no play makes Jack a dull boy,—and we have already too many Jacks and dull boys. Come and give yourself at least three days, or two days, of needed recreation. You will feel better for it and be better as a man and as a doctor. The programme will be very attractive. Make an effort, and you can get off at least two days. Come!

